# Characterisation of the role played by ELMO1, GPR141 and the intergenic polymorphism rs918980 in Fuchs' dystrophy in the Indian population

**DOI:** 10.1002/2211-5463.70006

**Published:** 2025-02-19

**Authors:** Susmita Sharma, Samar Kumar Basak, Sujata Das, Debasmita Pankaj Alone

**Affiliations:** ^1^ School of Biological Sciences National Institute of Science Education and Research (NISER) Bhubaneswar Khurda India; ^2^ Homi Bhabha National Institute (HBNI) Mumbai India; ^3^ Disha Eye Hospitals, Barrackpore Kolkata India; ^4^ LV Prasad Eye Institute Bhubaneswar India

**Keywords:** *ELMO1*, FECD animal model, genetic association, *GPR141*, intergenic SNP

## Abstract

Fuchs' endothelial corneal dystrophy (FECD) is the most common type of primary corneal dystrophy and can result in corneal transplantation. Here, we investigated the genetic association of SNP rs918980 (A>G) with FECD and the role of its surrounding genes *ELMO1* and *GPR141*. First, 128 patients and 379 controls were genotyped by Sanger sequencing. Our results show that rs918980 is significantly associated with FECD in the Indian population. Furthermore, *in silico* analysis suggested that rs918980 and its surrounding 150 bp region could regulate the transcriptional activities of nearby genes. Thus, we assessed whether *ELMO1* and *GPR141* were differentially expressed in FECD patients and in the corneal tissue of a UVA‐induced FECD mice model. Both genes were significantly upregulated and western blots studies concluded that protein levels of ELMO1 and GPR141 were also higher in the corneal endothelium of the UVA‐exposed eye compared to the control eye. Taken together, our results suggest that *ELMO1* and *GPR141* might play a significant role in FECD progression. However, further studies are required to better characterize the possible role of rs918980 and its nearby region in the regulation of *ELMO1* and *GPR141*.

## Graphical abstract

This study reports the upregulation of ELMO1 and GPR141 in human Fuchs' endothelial corneal dystrophy (FECD) corneal endothelium and ultraviolet A‐induced FECD mice model. A genetic association of an intergenic single nucleotide polymorphism rs918980 present between both genes is observed with FECD in the Indian population.

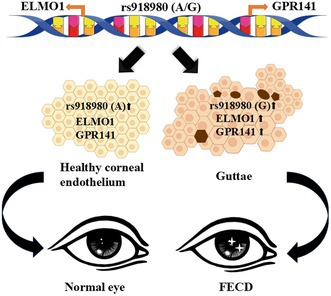

**Abbreviations**
CE: corneal endothelium
CEC: corneal endothelial cell
DSEK: Descemet stripping endothelial keratoplasty
ECM: extracellular matrix
EMT: epithelial–mesenchymal transition
EndoMT: endothelial–mesenchymal transition
FECD: Fuchs endothelial corneal dystrophy
IL: interleukin
LVPEI: LV Prasad Eye Institute
NFκB: nuclear factor kappa B
qRT‐PCR: quantitative real‐time PCR
SNP: single nucleotide polymorphism
TF: transcription factor
TGF: transforming growth factor
UVA: ultraviolet A

The innermost part of the human cornea, the corneal endothelium (CE), plays a vital role in vision and is present on the Descemet membrane [1]. CE consists of corneal endothelial cells (CECs), which are enriched with Na^+^‐K^+^ ATPase, aquaporin and other channel proteins that regulate the water and ions exchange between the corneal stroma and aqueous humor, keeping the avascular cornea in a hydrated condition and maintains corneal clarity [2, 3]. Dysregulation of these proteins leads to water retention in the corneal stroma, resulting in thickened Descemet's membrane, increased extracellular matrix (ECM) protein deposition, CECs loss, guttae formation, painful epithelial bullae leading to Fuchs' endothelial corneal dystrophy (FECD) [4, 5, 6, 7]. Cellular and molecular mechanisms such as endothelial pump dysfunction, endothelial–mesenchymal transition (EndoMT), RNA toxicity, oxidative stress, apoptosis, mitophagy and unfolded protein response pathway dysregulation contribute to the disease [8]. FECD, the most common type of primary corneal dystrophy, was first reported by Ernst Fuchs in 1910 and follows an autosomal dominant inheritance pattern in families. However, the number of sporadic cases is prominent and contributes to the significant number of corneal transplantation surgeries across the globe [9, 10, 11]. It has both early‐onset (< 40 years) and late‐onset (> 40 years) forms, with the latter being more common, showing female bias (male : female = 1 : 2) [12]. Based on the number of corneal guttae observed on CE, the disease is categorized on a severity scale of zero to four, with four being the most severe (advanced FECD condition with corneal edema) [13]. Disease prevalence varies with different populations, and a higher incidence was reported in individuals of Western and European descent, with 3.9% of the American population (> 40 years), 11.0% of the population of Tangier Island, US (> 50 years), and 11.0% of women and 7.0% of men are affected in Iceland [14, 15, 16]. The prevalence rate is 3.7% and 6.6% in the Japanese and Chinese Singaporean population respectively [17]. In India, 16.6% of penetrating keratoplasty surgery was performed between 1995 and 2001 for late‐onset FECD only in South India [18]. LV Prasad Eye Institute (LVPEI), Hyderabad, operated on 7.5–10.4% of FECD cases from 2007 to 2011 [19]. A single nucleotide polymorphism (SNP) rs613872 present in the third intron of the *Transcription factor 4* (*TCF4*) gene was found to be associated with the disease (*P =* 2.3 × 10^−26^) [20]. Further studies in individuals of Western descent with this gene showed that 79% of patients with FECD harbor a long stretch of > 50 CTG trinucleotide repeats in the third intron [21, 22]. Other populations worldwide showed the presence of CTG repeats in FECD cases but differed in their penetrance: 77.0% in Germans, 51.0% in Australians, 43.0% in Chinese, 26% in Japanese and 34.0% in Indians [23, 24, 25, 26, 27]. A genome‐wide association study showed *Laminin Subunit Gamma 1* (*LAMC1*) rs3768617, *KN Motif And Ankyrin Repeat Domains 4* (*KANK4*) rs79742895, and an intergenic, rs1200114 present between *ATPase subunit beta‐1* (*ATP1B1*) and *LINC00970* to be significantly associated with the late‐onset FECD disease in the Western population [28]. Recent studies from our laboratory showed that rs3768617 and rs1200118, which are in linkage disequilibrium with rs1200114, were significantly associated with the disease in the Indian population [29, 30]. *TCF4* mutations rs613872, rs17089887, rs17595731 and rs11659764 are also found to be significantly associated with FECD in the Indian population [26, 31, 32]. Genetic mutations in *COL8A2* are associated with early onset FECD, which is distinct from the late‐onset sporadic FECD. Genetic mutations in *SLC4A11*, *AGBL1*, *LOXHD1* and *DMPK* genes are also present in FECD patients [12]. Afshari *et al*. [33] performed a genome‐wide linkage screening with 92 FECD patients from 22 families and found that chromosomes 1, 7, 15, 17 and X showed high linkage signals. The multipoint analysis identified a few top marker SNPs in these linkage regions [33].

Penetrance levels and severity of FECD‐associated genetic mutations vary with ethnicity. Thus, we attempted to bridge the gap by finding new variations contributing to the genetic load of the late onset FECD in the Indian population. The present study assessed the genetic and functional association of a top marker intergenic SNP, rs918980, on chromosome 7, from the study undertaken by Afshari *et al*. [33]. We have also checked the role of the genes *ELMO1* and *GPR141*, which are adjacent to rs918980. This study reports the significant genetic association of the rs918980 with FECD in the Indian population, along with an upregulation of mRNA and protein levels of ELMO1 and GPR141.

## Materials and methods

### Sample collection

Sample collection was carried out in line with the principles of the Declaration of Helsinki after obtaining informed consent from the study participants at LVPEI, Bhubaneswar and Disha Eye Hospital, Barrackpore, Kolkata, with institutional approval from the ethics committee of both of the hospitals and NISER, Bhubaneswar (NISER/IEC/2022‐13). The consent was in written form with the signature of the participant. Four milliliters of blood was drawn from the FECD patients and the control individuals in EDTA vials and stored at −80 °C. The CE tissue samples from consented donors were collected from the stage 4 FECD patients with corneal edema who underwent Descemet Stripping Endothelial Keratoplasty (DSEK) surgery for visual rehabilitation and were stored in RNA later at −80 °C.

### Genomic DNA extraction and TaqMan genotyping

DNA extraction was performed using the salting out method from the collected blood samples [34]. The extracted DNA was kept at −80 °C for future use. Predesigned TaqMan® SNP Genotyping Assays for rs918980, rs66496742 and rs6975846 were purchased from Thermo Fisher Scientific (Waltham, MA, USA). Fifty nanograms of the extracted genomic DNA was directly subjected to the assay mix and TaqMan assay master mix (Applied Biosystems, Waltham, MA, USA) and genotyped in a Quant Studio™ 7 Flex Real‐Time PCR machine (Applied Biosystems).

### Confirmation of genotype by Sanger sequencing

Extracted genomic DNA from 20 random samples was subjected to PCR amplification using a pair of primers (Table S1), which amplifies the intergenic region surrounding rs918980, rs66496742 and rs6975846. The primer design was conducted using primer‐blast and purchased from GCC Oligos (Kolkata, India). The amplified product was processed for Sanger sequencing in a 3500xl Genetic Analyzer (Applied Biosystems) and the result was analyzed using bioedit program [35]. The sequencing result was matched with the data generated by the TaqMan genotyping to confirm the result.

### Cell culture

HEK293 cells were purchased from the National Centre for Cell Science (NCCS, Pune, India). The cells were cultured in Dulbecco's modified Eagle's medium, High glucose (HiMedia Laboratories Pvt. Ltd, Mumbai, India) with 10% fetal bovine serum (Gibco, Waltham, MA, USA), 5.0% antibiotic solution 100× (HiMedia Laboratories Pvt. Ltd) and 5.0% Amphotericin B solution (HiMedia Laboratories Pvt. Ltd) under 5.0% CO_2_ at 37.0 °C.

### Dual luciferase reporter assay

The dual‐luciferase reporter assay was employed to check the regulatory role of rs918980 wherein a region with 150 bp surrounding the SNP was PCR amplified by a pair of primers (Table S1) from two different patient samples having homozygous copies of alternate alleles. The amplified region was subjected to double restriction digestion with *Kpn*I (High Fidelity) and *Xho*I enzymes (New England Biolabs, Ipswich, MA, USA) to generate the sticky ends. This experiment was performed using the pGL4.23 firefly luciferase and the pGL4.74 renilla luciferase vectors (Promega, Madison, WI, USA) in accordance with the procedure reported previously [29]. The luciferase reporter activity of the constructs with alternate alleles of rs918980 was measured with a plate reader Varioskan lux (Thermo Fisher Scientific).

### *In silico* analysis

*In silico* analysis for the transcription factor (TF) prediction was performed using the JASPAR database (https://jaspar.genereg.net/). The 150 nucleotide sequences surrounding rs918980 with either allele ‘A’ or ‘G’ were analyzed to check whether any TF is binding to the region. The binding efficiency score was set to 100% in the JASPAR database search. The genomic position of the SNP was studied using the NCBI database and UCSC genome browser (https://genome.ucsc.edu/cgi‐bin/hgGateway, https://www.ncbi.nlm.nih.gov/snp/rs918980).

### Selection of tag SNPs

Tag SNP selection for rs918980 was performed with the Ensembl genome browser (https://www.ensembl.org/index.html) with pairwise *r* ^2^ and *D*′ value = 1, which shows that the tag SNP is in strong linkage disequilibrium with the lead SNP (https://asia.ensembl.org/index.html). Two tag SNPs, rs6974846 and rs66496742, were selected for further study.

### Quantitative real‐time PCR (qRT‐PCR)

Preserved control and FECD tissue samples were thawed, RNA was extracted using RNeasy Mini Kit (QIAGEN, Hilden, Germany) and immediate conversion into cDNA was achieved using the verso cDNA synthesis kit (Thermo Fisher Scientific). The qRT‐PCR was performed with 5 ng of cDNA and 0.4 μm forward and reverse primer pairs for *GPR141* and *ELMO1* (Table S1). β‐Actin was used as an internal control. The reactions were performed using Fast Start Universal SYBR green master mix (Thermo Fisher Scientific) in a QuantStudio™ 5 Real‐Time PCR System (Applied Biosystems) and fold change was calculated by applying the $2^{-\Delta \Delta C_{t}}$ method. Changes in gene expression levels were determined after normalizing the values with internal control, β‐actin.

### Immunofluorescence

The control and patient tissue samples from Disha Eye Hospital were fixed using 4.0% paraformaldehyde, then permeabilized using phosphate‐buffered saline with 0.5% Triton X‐100 and blocked using 10.0% normal horse serum. The tissue samples were incubated overnight with primary antibodies of ELMO1 (sc‐271519; dilution 1 : 500) or GPR141 (DF‐2797; dilution 1 : 200) at 4.0 °C. Samples were incubated with Alexa Flour conjugated secondary antibody (dilution 1 : 500; Invitrogen, Waltham, MA, USA) for 1 h at room temperature. The counterstaining was subsequently performed using DAPI (i.e. 4′,6‐diamidine‐2′‐phenylindole dihydrochloride) (Sigma, St Louis, MO, USA). Then, tissue was mounted on the slide using Fluoromount‐G (Southern Biotech, Birminghma, AL, USA). After drying, the slides were imaged using SP8 DLS Confocal Microscope (Leica, Wetzlar, Germany) and processed using imagej (nih, bethesda, md, usa).

### Animal model regeneration and tissue collection

Experiments involving animals were performed after obtaining the ethical approval from the Institutional Animal Ethics Committee, NISER (NISER/SBS/AH‐268). FECD animal model development by ultraviolet‐A (UVA) induction was performed as described by Liu *et al*. [36]. In total, 10 female C57BL/6J mice aged 7–12 weeks were exposed to UVA radiation with a fluence of 500 J·cm^−2^ (irradiance = 368 mW·cm^−2^, time = 23 min). The left eye was used for the UVA irradiation and the right eye of the same mouse was used as the control. The animals were kept under observation for 90 days. Slit lamp microscopy images were taken at pre‐UVA exposure, day 1, 30, 60 and 90 post‐exposure. At the end of 3 months, the mice were killed by inhalation of carbon dioxide in a euthanasia chamber, and corneal cup tissues were collected. CE was removed surgically from the corneal cup. The tissues were stored in RNA later at −80 °C. The mice were used in this experiment as per the ARVO Statement for the Use of Animals in Ophthalmic and Vision Research.

### Western blotting

Corneal endothelial tissue from the mice's control and UVA‐exposed eye was homogenized in ice using radioimmunoprecipitation buffer, the supernatant was collected, and protein concentration was quantified using the Bradford assay. Twenty‐five micrograms of the denatured protein was loaded into each well for 12.0% SDS/PAGE. Then, the protein was transferred to the poly(vinylidene difluoride) membrane (IPVH00010; Millipore, Burlington, MA, USA) using the wet transfer method at 50 V for 6.0 h. The membrane was then blocked by 5.0% skim milk dissolved in 1× Tris‐buffered saline with 0.1% Tween 20. Primary antibody incubation was carried out overnight with ELMO1 (sc‐271519; dilution 1 : 1000), GPR141 (DF‐2797; dilution 1 : 500) and β‐actin (8H10D10; dilution 1 : 1000). Horseradish peroxidase‐conjugated goat anti‐mouse (621140680011730; dilution 1 : 5000) and goat anti‐rabbit IgG (621140380011730; dilution 1 : 5000) were used as a secondary antibody. Chemiluminescence was detected using a Super Signal Femto Maximum Sensitivity Substrate kit (PI34094; Thermo Fisher Scientific) in a Chemidoc MP system (Bio‐Rad, Hercules, CA, USA). Obtained bands were analyzed using image lab (Bio‐Rad). The protein expression levels were measured relative to β‐actin endogenous control levels.

### Statistical analysis

Statistical analysis with genotyping data was conducted using spss, version 20.0 software (IBM Corp., Armonk, NY, USA). A ch‐squared test and a 10 000 permutation test were carried out to calculate the allelic frequency difference between the two groups, and the genetic association was performed using haploview, version 4.2 (Broad Institute, Cambridge, MA, USA). Statistical power calculation for the study was performed using the Online Sample Size Estimator (osse) tool (http://osse.bii.a‐star.edu.sg/calculation1.php).

## Results

### An intergenic variant rs918980 present between *ELMO1* and *GPR141* gene showed a positive genetic association with FECD in the Indian population

For assessing the genetic association, 128 FECD and 379 age and sex‐matched control participants were selected from the recruited participants at LVPEI, Bhubaneswar and Disha Eye Hospital, Barrackpore. The study participants of both groups were of the same age group with a *P* value of 0.36 (*P* > 0.05). The control and patient groups had a male‐to‐female ratio of 1 : 1.78 and 1 : 2.15, respectively, aiming to avoid any gender bias in the study. The demographic data of the study groups are provided in Table S2. Intergenic SNP rs918980 [chr7:37562295 G>A, (GRCh38.p14)] is present between two genes: *ELMO1* (MIM:606420) and *GPR141* (MIM:609045) (Fig. S1). The SNP is present near the 5′ UTR region of both the genes. Tag SNPs rs6975846 [chr7:37558680 G>C, (GRCh38.p14)] and rs66496742 [chr7:37558746 G>C, (GRCh38.p14)] are present at 3614 and 3549 bases upstream of the lead SNP rs918980.

The genetic association of this SNP with FECD was determined by calculating the allelic frequency difference between the control and the FECD study participants. It was found that rs918980 is significantly associated with FECD cases in the Indian population with a chi‐square value of 4.97 and a *P* value of 0.01 (odds ratio = 1.41; 95% confidence interval = 1.02–1.56) and the ‘G’ allele emerged as the risk allele for the disease condition. The 10 000 permutation test showed the significant association of the SNP with the disease remained strong (*P* = 0.03 and χ^2^ = 4.91). The allele ‘G’ also exhibited as a risk with the dominant, recessive and additive genetic models. The statistical power of this association study is 30.9%. The result indicates that the presence of the ‘G’ allele in the Indian population increases the risk for FECD.

For rs66496742, the study showed that the allelic frequency difference between the control and FECD participants is not significant (*P* = 0.52, odds ratio = 0.99; 95% confidence interval = 0.71–1.39; 10 000 permutations, *P* = 1.00). Analysis with different genetic models did not show any significant association of the SNP with FECD. The statistical power is at 2.5%. Similarly, statistical analysis of rs6975846 genotype data through different genetic association models, such as the allelic model, dominant model, recessive model and additive model, did not show any significant association with the FECD with a statistical power of 3.2%. Genetic model analysis for all three SNPs is shown in Table 1.

**Table 1:** Genetic association analysis of rs918980 [chr7:37562295 G>A, (GRCh38.p14)] and its tag SNPs rs66496742 [chr7:37558746 G>C, (GRCh38.p14)] and rs6975846 [chr7:37558680 G>C, (GRCh38.p14)] with FECD in the Indian population.

SNP ID	Type		Control count (frequency) (*N* = 379)	FECD count (frequency) (*N* = 128)	Model	χ^2^ value	*P* value	Odds ratio (95% confidence interval)
rs918980	Allele	A	280 (0.36)	75 (0.29)	Allelic	4.97	**0.01**	**1.41 (1.02–1.56)**
		**G**	478 (0.63)	181 (0.70)				
	Genotype	AA	65 (0.17)	12 (0.09)	Dominant	4.49	**0.02**	**0.50 (0.26–0.95)**
		AG	150 (0.39)	51 (0.40)	Recessive	2.17	0.08	1.35 (0.90–2.02)
		**GG**	164 (0.43)	65 (0.50)	Additive	2.83	0.06	1.81 (0.90–3.64)
						0.28	0.33	0.59 (0.56–1.38)
rs66496742	Allele	C	185 (0.25)	62 (0.25)	Allelic	0.001	0.52	0.99 (0.71–1.39)
		G	527 (0.74)	178 (0.74)				
	Genotype	CC	29 (0.08)	9 (0.07)	Dominant	0.05	0.49	0.91 (0.41–1.98)
		CG	127 (0.35)	44 (0.36)	Recessive	0.009	0.50	0.98 (0.64–1.48)
		GG	200 (0.56)	67 (0.55)	Additive	0.03	0.47	1.04 (0.67–1.62)
						0.03	0.51	0.92 (0.41–2.05)
rs6975846	Allele	C	235 (0.33)	77 (0.32)	Allelic	0.06	0.43	1.04 (0.76–1.42)
		G	477 (0.66)	163 (0.67)				
	Genotype	CC	52 (0.14)	12 (0.10)	Dominant	1.66	0.12	0.64 (0.33–1.25)
		CG	131 (0.36)	53 (0.44)	Recessive	0.30	0.32	0.89 (0.58–1.34)
		GG	173 (0.48)	55 (0.45)	Additive	1.22	0.16	1.28 (0.82–1.99)
						0.81	0.23	0.72 (0.36–1.45)

*Note*: Bold letters indicate risk allele and the risk genotype, bold values indicate (*P* < 0.005).

### rs918980 is present in a regulatory intergenic region

**Fig. 1:**
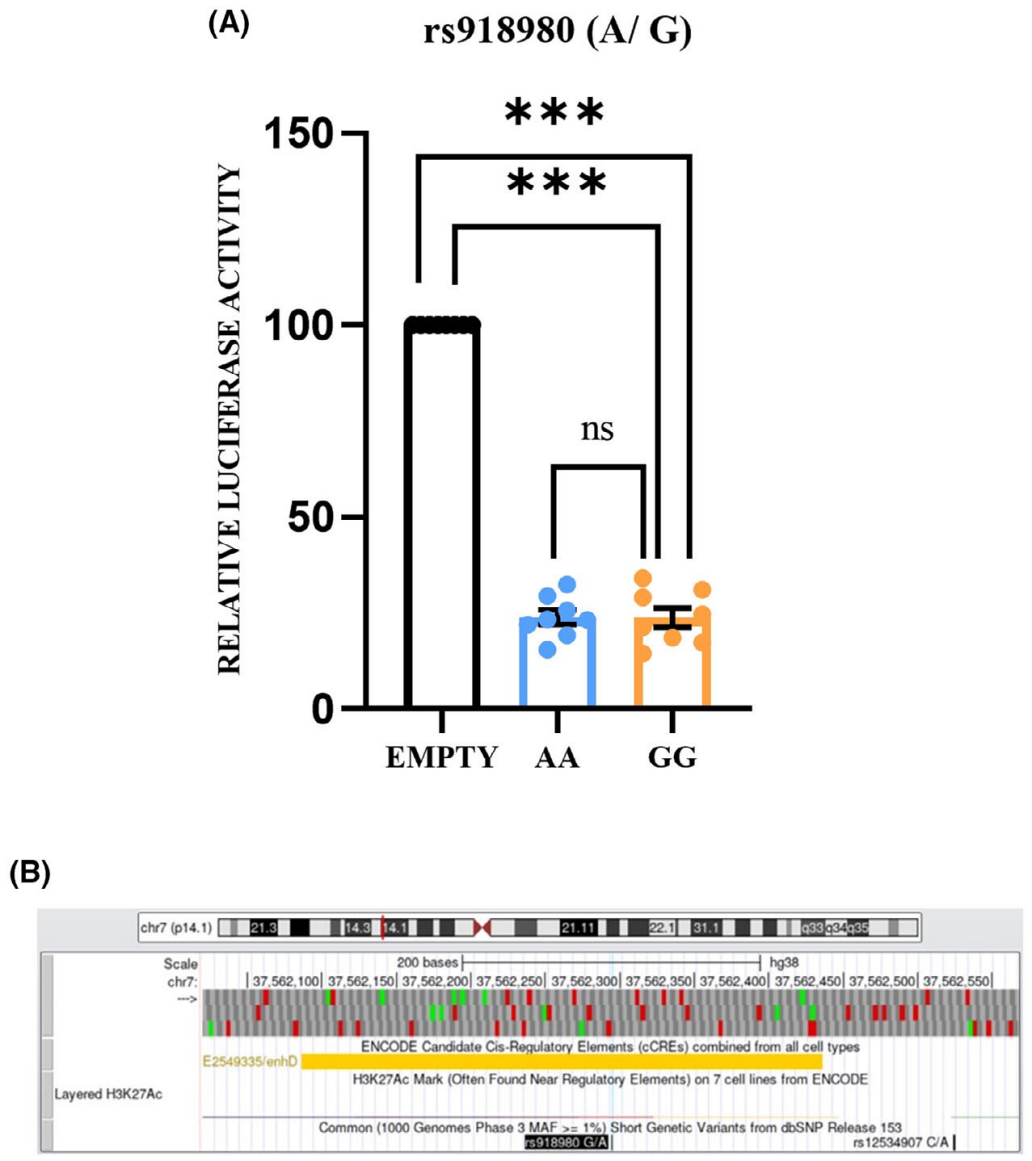
(A) Relative luciferase activity of 150 bp region surrounding the rs918980. A Dual‐luciferase reporter assay was performed to examine the function of allelic transition in the case of rs918980, i.e., from ‘A’ to ‘G’. A significant difference in the reporter activity was measured between the empty vector and construct with allele ‘A’ and ‘G’. The luciferase activity remained unchanged with the allele ‘A’ transition to ‘G’. Student *t*‐test was carried out to calculate the statistical significance. ***P < 0.0001; ns, non‐significant, *P* > 0.05. The values are presented as the mean ± SEM. (B) The genomic location of rs918980 and its nearby region per hg38. The UCSC genome browser shows that the 150 bp surrounding the SNP rs918980 are on a *cis*‐regulatory element (CRE) and have an H3K27Ac signal. The yellow color denotes that the CRE can act as a distal enhancer.

A dual luciferase reporter assay was performed to check the regulatory role of rs918980 where a 150 bp region flanking the SNP was PCR amplified and cloned into a luciferase pGL4.23 vector, either with allele ‘A’ or ‘G’. The result showed significant downregulation in luciferase activity in comparison to the empty vector with a vector with allele ‘A’ (23.82 ± 10.8, *P* = 1.4 × 10^−5^) or ‘G’ (23.76 ± 11.7, *P* = 1.2 × 10^−5^) (Fig. 1A), indicating that the intergenic region has regulatory property. However, the luciferase activity difference remained non‐significant with the change in allele from ‘A’ to ‘G’ (*P* = 0.94), suggesting the activity of the intergenic region is not biased by the allelic transition. The experiment was performed on three separate days in triplicate for each construct, and the obtained luminometric readings are represented as the mean ± SEM.

### Binding of transcription factors leads to the regulatory activity of the 150 bp genomic region irrespective of allele ‘A’ or ‘G’

The UCSC genome browser search identified rs918980 and its surrounding 150 bp region as present in a *cis*‐regulatory element with a distal enhancer‐like signature and is near the H3K27ac mark, which indicated that the region could regulate the transcriptional activities of nearby genes (Fig. 1B). *In silico* analysis with the JASPAR database search showed that nine TFs bind to the 150 bp region flanking rs918980 with 100% efficiency (Table S3). Yet, no specific TF binds differentially to only allele ‘A’ or ‘G’, showing that the regulatory activity displayed by this region may not be allele‐specific.

### Significant upregulation of ELMO1 mRNA and protein in FECD patients and the corneal tissue of the UVA‐induced FECD mice model

**Fig. 2:**
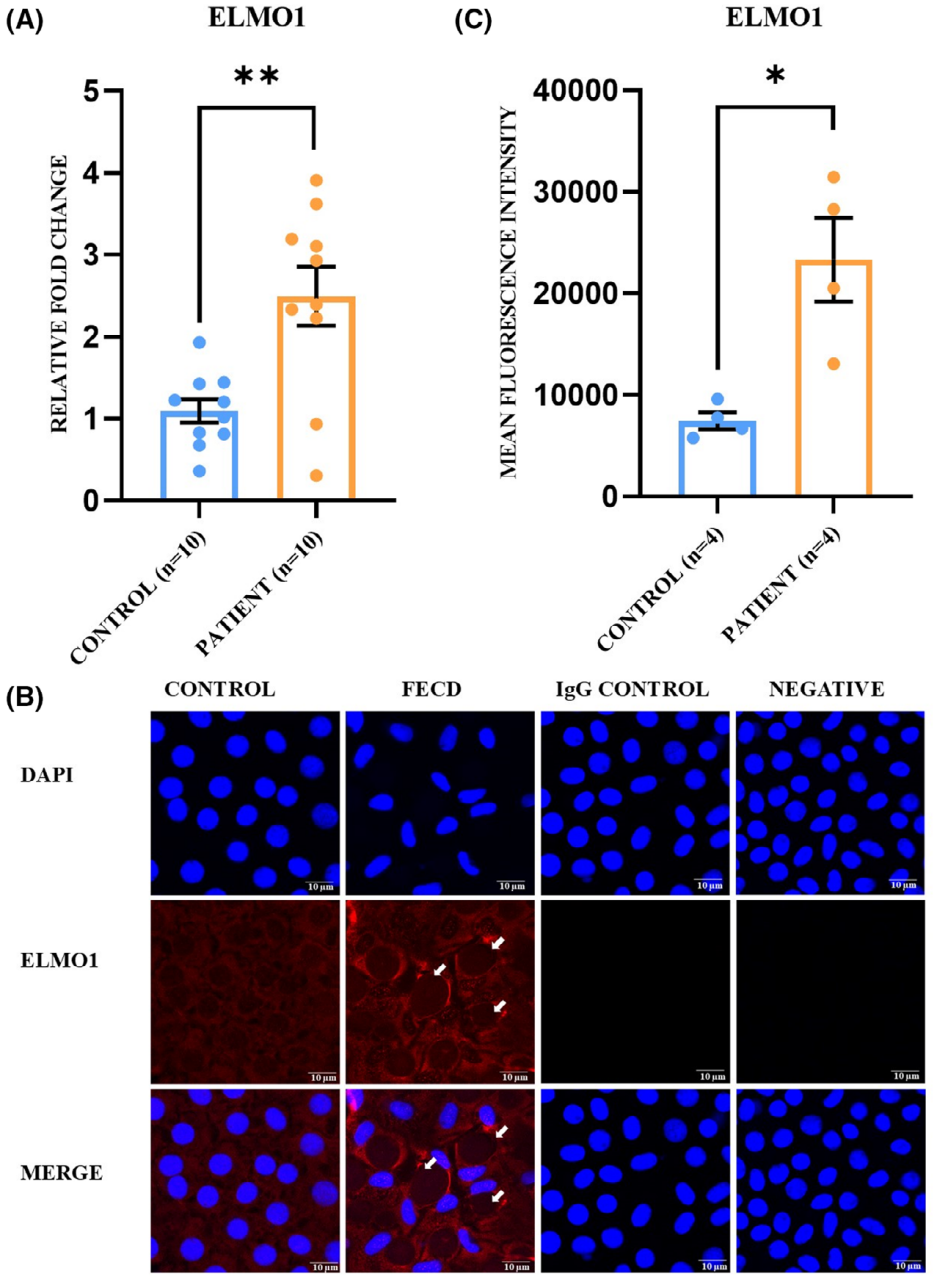
Increased mRNA and protein expression of ELMO1 in human FECD sample. (A) qRT‐PCR analysis revealed that mRNA expression of *ELMO1* is significantly increased in case of FECD patients. Statistical significance was calculated using a *t*‐test [**P = 0.003, control (*n* = 10), patient (*n* = 10)] and the obtained values are noted as the mean ± SEM. (B) Immunofluorescence experiment with ELMO1 revealed overexpression of the protein in FECD corneal endothelium. The presence of guttae on the endothelium tissue showed the severity of the disease (white arrow). A minimal background signal was observed in the case of rabbit isotype control (green), and no signal was observed in the case of mouse isotype control (red). A negative control experiment was performed without the primary antibody, and no signal was obtained in the case of ELMO1, ensuring the specificity of the obtained signal with the primary antibody. (C) Quantification of ELMO1 immunofluorescence signal revealed a significant increase in its protein level [*P = 0.02, control (*n* = 4), patient (*n* = 4)] in FECD with a *t*‐test. The corrected total cell fluorescence (CTCF) value is represented as the mean ± SEM. Magnification = 630×, zoom factor = 2×, scale bar = 10 μm.

**Fig. 3:**
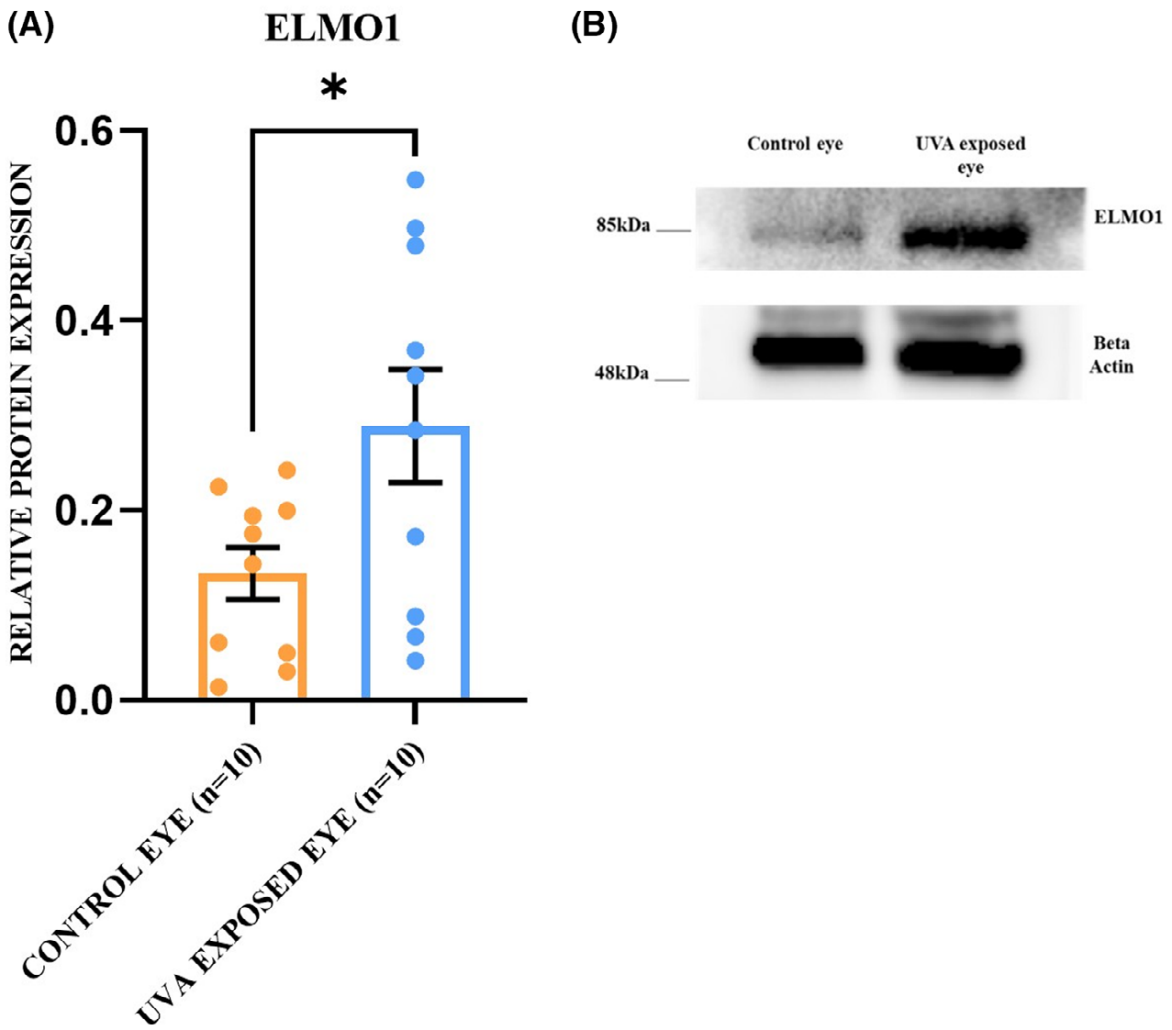
Increased protein expression of ELMO1 in UVA exposed eye of FECD mice model. (A) Western blot with ELMO1 in corneal endothelium tissue from control (*n* = 10) and UVA‐exposed eye (*n* = 10). β‐Actin was taken as the internal control and used to normalize the obtained ELMO1 value. (B) Quantification of the obtained bands showed significant upregulation of ELMO1 in the case of UVA‐exposed eyes (*P* = 0.03). The experiments were repeated three times on different days, and the obtained values are noted as the mean ± SEM. Statistical significance was calculated by a *t*‐test (*P < 0.05).

The role of the ELMO1 gene in FECD pathogenesis is yet to be explored. Because the intergenic region has distal enhancer‐like activity, we hypothesized that it may regulate the expression of nearby located genes. Accordingly, we performed the qRT‐PCR experiment to check the change in mRNA level of *ELMO1* (present 112 969 bp upstream to rs918980) in the FECD corneal endothelium. It was found that *ELMO1* mRNA expression is upregulated by 2.28‐fold in FECD (2.28 ± 0.32) cases compared to the controls (1 ± 0.13, *P* = 0.003) (Fig. 2A). Alteration in the protein expression level of ELMO1 was checked through the immunofluorescence method by taking four control and four patient DSEK tissues. ELMO1 is localized in the cytoplasmic region and is upregulated by 3.12‐fold in diseased endothelium (3.12 ± 0.17) compared to the control (1 ± 0.1, *P* = 0.02) (Fig. 2B,C). Western blot analysis was performed to check the change in protein expression of ELMO1 in the corneal endothelium tissue of the UVA‐induced FECD mice model. The protein level of ELMO1 was found to be significantly upregulated by 2.16‐fold in the UVA‐exposed eye (2.16 ± 0.20) compared to the control eye (1 ± 0.20, *P* = 0.03) (Fig. 3).

### Higher level of GPR141 mRNA and protein in human FECD corneal tissue and UVA‐exposed eye of the FECD mice model

**Fig. 4:**
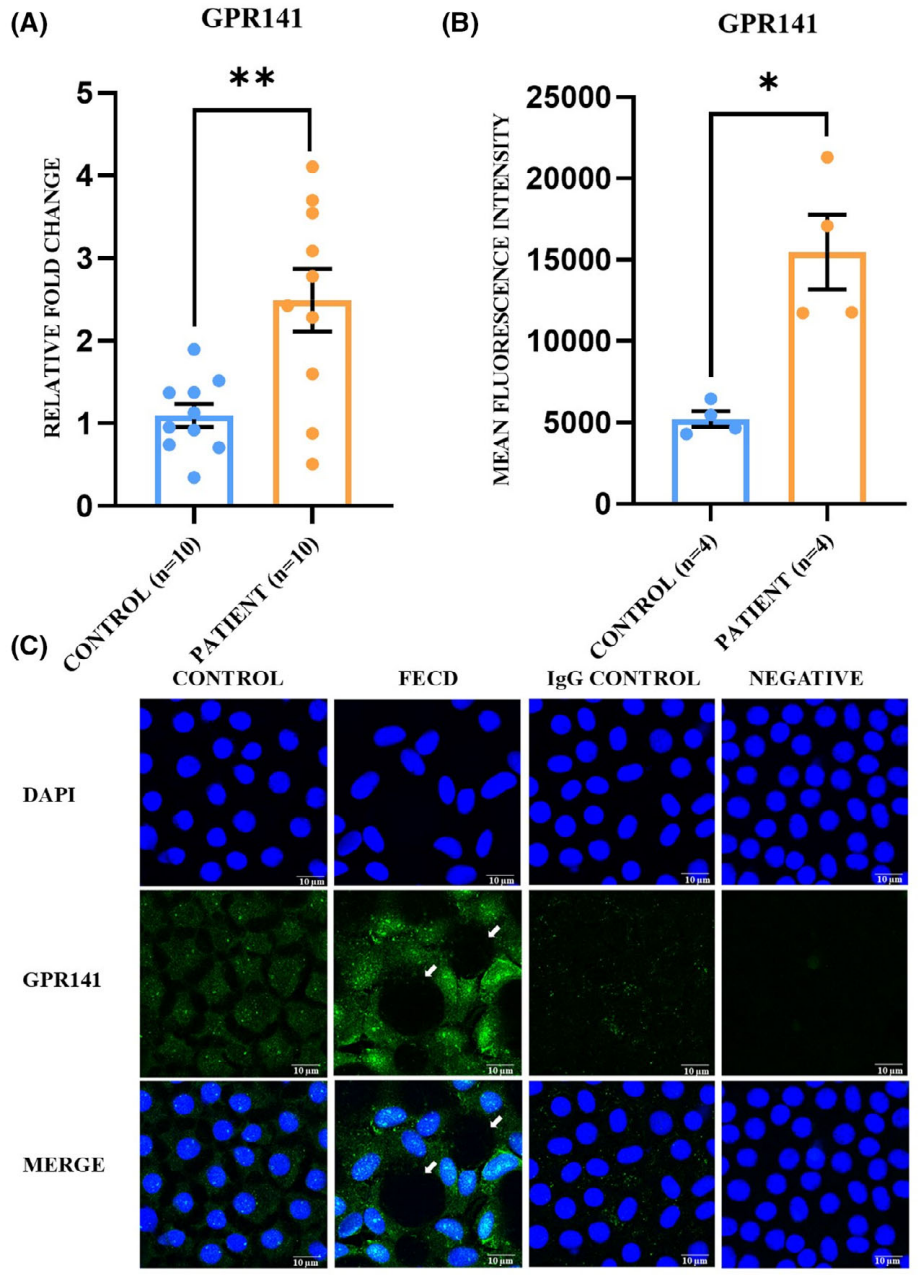
Increased *GPR141* mRNA and protein expression in human FECD tissue sample. (A) *GPR141* mRNA is significantly upregulated in disease condition [**P = 0.005, control (*n* = 10), patient (*n* = 10)]. The experiment with each gene was repeated three times on three different days, and the obtained values are noted as the mean ± SEM. Statistical significance was calculated by a *t*‐test. (B) Immunofluorescence shows that the GPR141 protein significantly increases in disease conditions in human DSEK tissue [*P = 0.01, control (*n* = 4), patient (*n* = 4)]. Statistical significance was calculated by a *t*‐test and the obtained CTCF value is represented as the mean ± SEM. (C) The presence of guttae on the endothelium tissue showed the severity of the disease (white arrow). Magnification = 630×, zoom factor = 2×, scale bar = 10 μm.

**Fig. 5:**
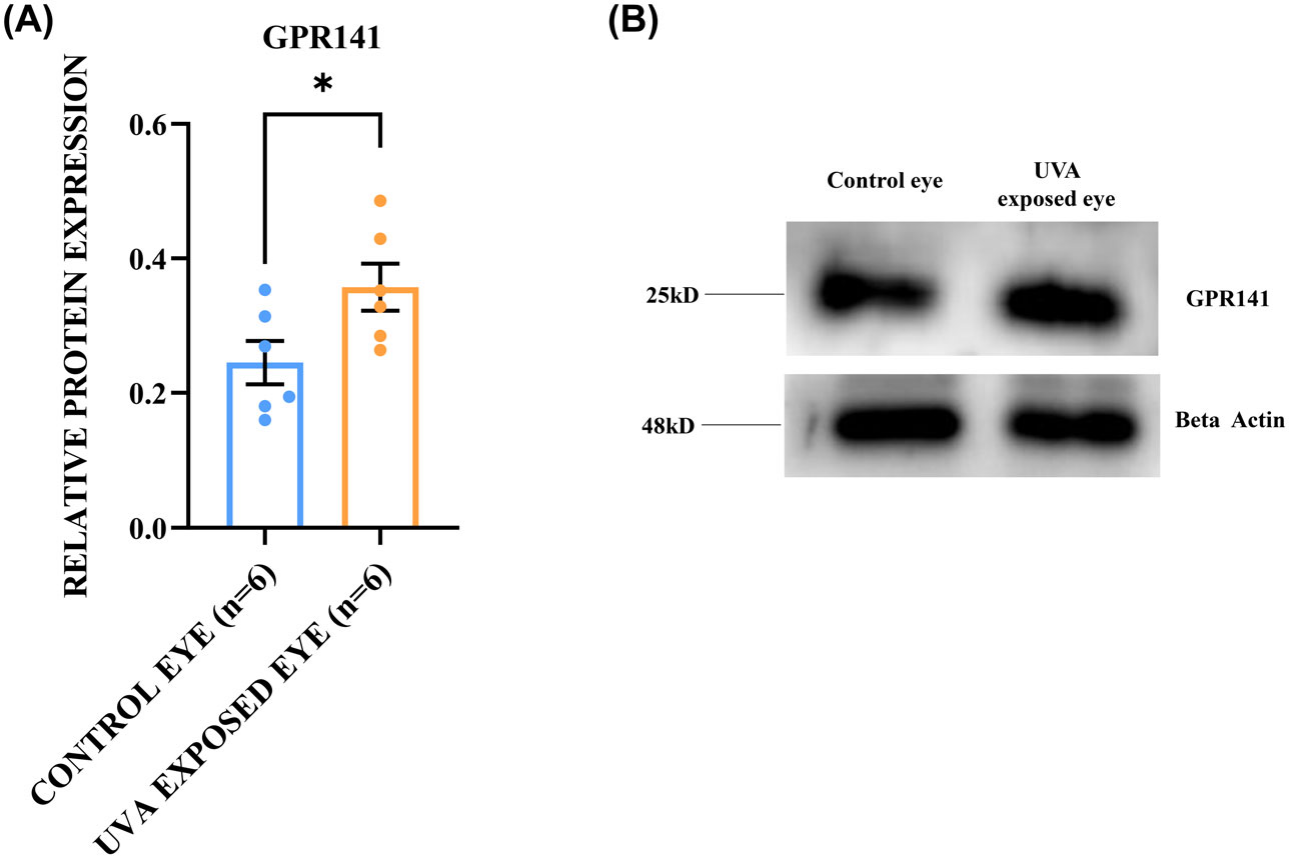
Increased protein expression of GPR141 in UVA exposed eye of FECD mice model and the proposed pathway of ELMO1 and GPR141 regulation in corneal endothelium. (A) Western was performed with GPR141 antibody in corneal endothelium from control (*n* = 6) and UVA‐exposed eye (*n* = 6) of mice. (B) Quantification showed significant upregulation of GPR141 protein level in UVA‐exposed eyes of mice (*P = 0.03). The experiments were repeated three times on different days, and the obtained values are noted as the mean ± SEM. Statistical significance was calculated by *t*‐test.

The function of the *GPR141* gene in FECD pathogenesis has not been studied to date. The qRT‐PCR experiment was performed to check the changes in *GPR141* mRNA level in FECD corneal endothelium. The result showed a 2.27‐fold increase in the mRNA level of *GPR141* in FECD corneal endothelium (2.27 ± 0.34) compared to healthy tissue (1 ± 0.13, *P* = 0.005) (Fig. 4A). The GPR141 protein level alteration was checked in the FECD corneal endothelial tissue from humans using the immunofluorescence technique. GPR141 is found to be present in both cytoplasm and nucleus, and its expression is significantly upregulated by 2.96‐fold in FECD tissue (2.96.46 ± 0.14) compared to healthy control tissue (1 ± 0.09, *P* = 0.01) (Fig. 4B,C). A western blot analysis was perfomred with the protein from the corneal endothelium of the UVA‐exposed eye and the normal eye of mice to check for any alteration in the GPR141 level. GPR141 showed a significant upregulation in its protein level in the UVA‐exposed eye (1.45 ± 0.09) by 1.45‐fold compared to the unexposed eye (1 ± 0.13, *P* = 0.03) (Fig. 5).

## Discussion

An intergenic region may contain several regulatory areas, such as promotors, enhancers and regulatory elements. Non‐coding intergenic disease‐associated SNPs are present near these regions and interact with their host regulatory elements to deliver the function [37]. The present study finds a positive genetic association of an intergenic SNP rs918980 with FECD in an Indian population with the ‘G’ as a risk allele, and individuals inheriting genotypes ‘AG’ and ‘GG’ have higher disease susceptibility. With FECD being a rare disorder, there is a limitation with the number of patient samples used in the study. However, to overcome this sample size limitation, we conducted a 10 000 permutation test, which resulted in a significant association of the SNP with the disease with a *P* value of 0.03. Follow‐up studies in larger cohorts and different populations will aim to validate this finding. The SNP is positioned at 7:37562295 (GRCh38) with major allele ‘G’ (0.56) and minor allele ‘A’ (0.43) in a group from the Indian population (Gujrati Indian in Houston) (https://asia.ensembl.org/Homo_sapiens/Variation/Population?db=core;r=7:37561795‐37562795;v=rs918980;vdb=variation;vf=729205638). We did not find any allele‐specific TF binding to rs918980 in the JASPAR database search. This result is corroborated by the luciferase assay result, which showed that the relative luciferase activity significantly changed with the presence of the 150 bp flanking region irrespective of the presence of allele ‘A’ or ‘G’ of the SNP.

GTEx analysis showed rs918980 affects the expression profile of nearby genes (i.e. *NME8*) (https://gtexportal.org/home/snp/rs918980). The SNP is present at a *cis*‐regulatory element, namely a distal enhancer‐like signature, representing a possible DNAase hypersensitivity site. This region has a histone acetylation mark (H3K27ac), which can enhance the transcription of nearby genes. Bioinformatic analysis showed two genes (i.e. *ELMO1* and *GPR141*) surrounding rs918980, which are present at a distance of 1.12 and 1.21 Mb, respectively. The study found overexpressed mRNAs and proteins for both genes in the case of human FECD corneal endothelium. This result was further supported by increased levels of both ELMO1 and GPR141 proteins in the diseased eye of the UVA‐induced FECD mice model.

*ELMO1* codes for human *Engulfment and cell motility protein 1*, which plays a vital role in cytoskeletal rearrangements during cellular movement, morphology change and phagocytosis of apoptotic cells [38]. During the normal developmental process, *ELMO1* is involved in cell clearance by apoptosis and maintains the homeostasis of tissues. It interacts with DOCK180 (i.e. Dedicator of cytokinesis 180) and activates Rac1, resulting in the actin cytoskeleton rearrangement, leading to the engulfment of apoptotic cells by macrophages [39, 40]. Various genetic and epigenetic changes in *ELMO1* leading to its overexpression have been observed in several diseases. It is found to be associated with tumor cell invasion and metastasis. It regulates different cytokine, chemokine and growth factor activation pathways in various cancer types [41].

With pathogenic bacterial infections, the ELMO1 initiates cytoskeletal rearrangement and producing proinflammatory cytokines such as tumor necrosis factor α [42]. Elevated tumor necrosis factor α levels were associated with reduced endothelial cell density and increased levels of interleukin (IL)‐6 and IL‐8. Prolonged exposure to higher cytokine levels generates intracellular reactive oxygen species, leading to ER stress, cell death and fewer endothelial cells [43, 44]. The levels of IL‐6 and IL‐8 were found to be elevated in the AH samples of FECD patients [45]. It remains to be seen if elevated levels of ELMO1 are responsible for elevated cytokines levels in FECD, leading to subsequent pathologies. Also, during *Salmonella* infection, *ELMO1* stimulates LC3II‐associated autophagy [46]. LC3II increases in FECD, leading to loss of mitochondria, indicating a possible role of *ELMO1* in mitophagy [47]. ELMO1 upregulation alters the renal transforming growth factor (TGF) β1 in diabetic nephropathy and lowers the TGFβ level, leading to anorectal cancer invasion [48, 49, 50]. By contrast to the previous findings obtained with respect to cancer, the increased expression level of TGFβ isoforms in FECD induces excessive ECM deposition, triggering the intrinsic apoptosis pathway [51]. Our study resulted in the upregulation of ELMO1 in FECD cases, which may act upstream of TGFβ signaling. However, the exact mechanism of TGFβ upregulation via ELMO1 in the case of FECD is yet to be revealed.

*ELMO1* acts as a significant regulator of EMT in different types of cancers through the IL‐8/ELMO1/nuclear factor kappa B (NFκB)/Snail pathway. Overexpression of IL‐8 leads to an increased level of *ELMO1*, which activates Snail protein, resulting in the overproduction of EMT markers [41]. A similar mechanism was reported in FECD, where the CECs lose contact with the neighboring cells and transform into fibroblast type, also known as EndoMT [52]. Studies have found upregulation of IL‐8, Snail and EMT markers such as ZEB1 and E‐cadherin in FECD [53, 54]. Correlating the previous results with those of the present study reveals that *ELMO1* might be acting as a major player in the EndoMT regulation in FECD because elevated ELMO1 level enhances Snail expression via the NFκB pathway leading to overproduction of EMT/EndoMT marker proteins. Also, the interaction of ELMO1 with SOX10 regulates the EndoMT by overexpression of Snail via activation of the phosphoinositide 3‐kinase/Akt pathway [41, 55, 56]. Accordingly, *ELMO1* may act as a regulator in phosphoinositide 3‐kinase/Akt, TGF‐β and NFκB signaling pathways leading to the FECD condition. IL‐8 stimulation and Snail overexpression also increase Rac1 expression, which leads to increased expression of matrix metalloproteases such as *MMP2* and *MMP9* to balance the dysregulation of ECM homeostasis [41, 55, 56]. Both of the MMPs are found to be upregulated in the case of FECD [57]. *ELMO1* mRNA overexpression was also found in various cancers [58, 59, 60]. *In silico* analysis via miRDB (https://mirdb.org) revealed that the *ELMO1* promotor has a binding site for a microRNA hsa‐miR‐30b‐3p. This particular miRNA is found to be hypermethylated in the case of FECD [58]. Because miRNA acts to silence the target gene, hypermethylation of the miRNA in FECD can lead to its downregulation and, ultimately, the overexpression of its target gene *ELMO1*.

*GPR141* belongs to the Class A orphan receptor of the rhodopsin family. Different G protein‐coupled receptors have a role in cell invasion, proliferation, formation of blood vessels, and chemoresistance during tumor development and progression [59, 60, 61]. *GPR141* was found to be overexpressed in breast cancer and downregulated of p53 level by mammalian target of rapamycin/p53 axis and elevates a primary EMT marker such as Snail as found in case of FECD [59, 62]. The level of p53 was found to be upregulated in FECD, playing an important role in oxidative stress‐induced apoptosis of CECs [62]. Further study in FECD can reveal how the mammalian target of rapamycin/p53 axis is regulated by *GPR141* overexpression in the disease condition. Additionally, it was found that, in inflammatory breast cancer cells, both the *ELMO1* and *GPR141* mRNA and protein levels were upregulated, which increases the activity of RhoC, a cytoskeletal remodeling protein leading to actin polymerization helping in the migration of inflammatory breast cancer cells [63, 64]. *GPR141* might be crucial in cytoskeletal rearrangement during cell size and shape changes, as well as apoptotic cell clearance in FECD.

**Fig. 6:**
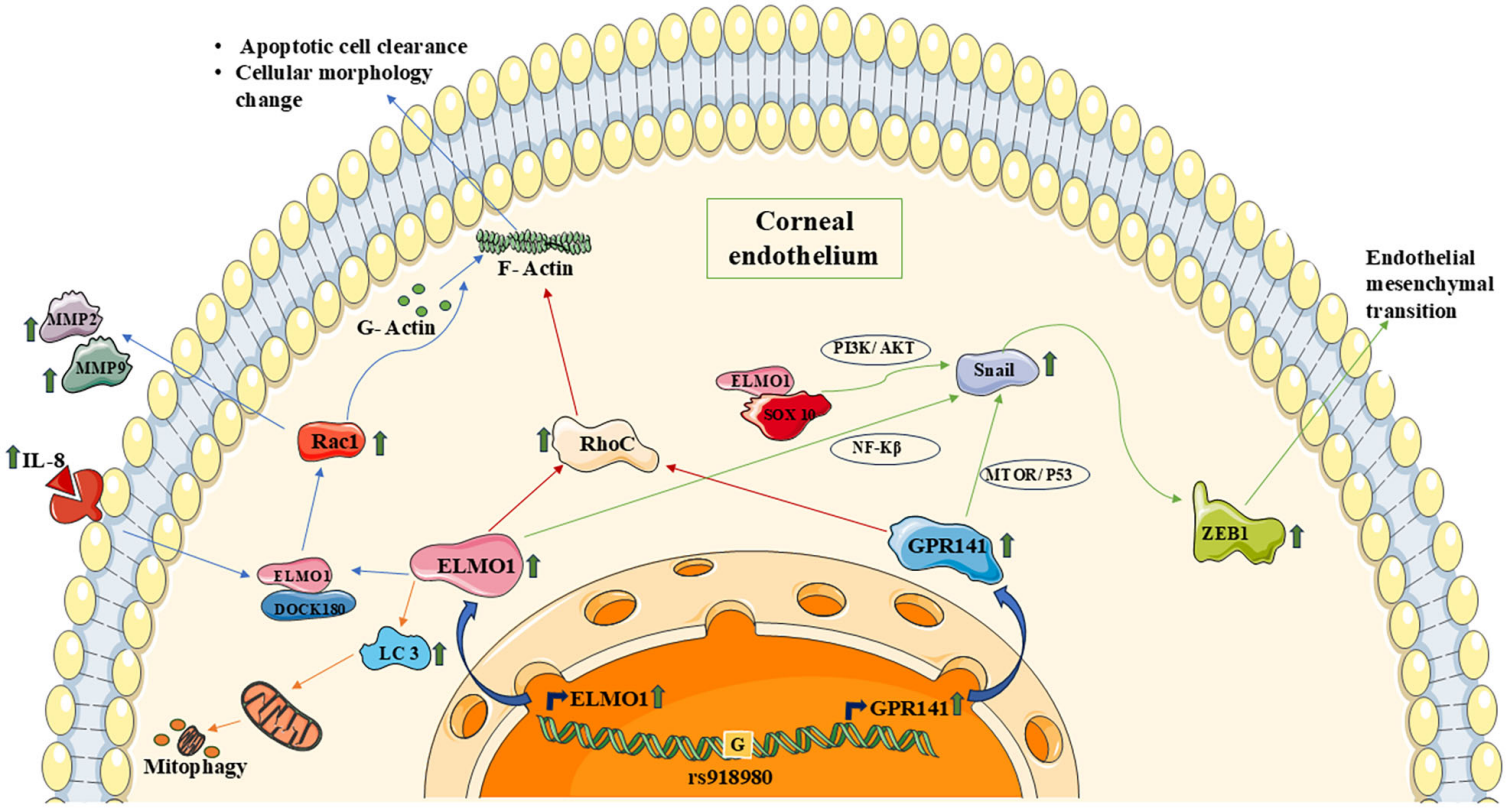
Proposed pathway showing the mechanism of GPR141 and ELMO1 in corneal endothelium leading to FECD pathogenesis and progression. ELMO1 and GPR141 regulate the changes in cellular morphology, cell death, mitophagy and EndoMT through various pathways. Ceated using Servier Medical Art (https://smart.servier.com).

Previous research findings link this study result with reactive oxygen species generation as a result of UVA exposure, leading to the FECD disease phenotype. The above study indicates *ELMO1* and *GPR141* might play a significant role in FECD progression, acting in various pathways, such as the EndoMT and actin polymerization responsible for the morphological alteration of CECs, apoptosis and autophagy (Fig. 6). Future studies will reveal whether there is any actual interaction between the intergenic region and the promotors of both genes regulating their expression.

## Conclusions

We conclude with the contribution of rs918980 to the FECD in the Indian population. Increased *ELMO1* and *GPR141* mRNA and protein levels were found in FECD individuals. Furthermmore, the present study was strengthened by increased mRNA and protein levels of both of the genes in the UVA‐induced FECD mice model, indicating their role in FECD disease pathogenesis and progression.

## Conflicts of interest

The authors declare that they have no conflicts of interest.

### Peer review

The peer review history for this article is available at https://www.webofscience.com/api/gateway/wos/peer‐review/10.1002/2211‐5463.70006.

## Author contributions

SS contributed to the investigation, methodology, formal analysis of data and writing the original draft. SKB provided the samples, acquired the clinical data and collected informed consent from study participants (Clinical Collaborator). SD also provided us with samples, acquired clinical data and collected informed consent from study participants (Clinical Collaborator). DPA contributed towards study conceptualization, funding acquisition, project administration, and supervision, and helped in reviewing and editing the original draft.

## Supporting information


**Fig. S1.** Genomic location of SNPs rs918980, rs66496742 and rs6975846.
**Table S1.** List of primers.
**Table S2.** Demographics of the study participants in the genetic association studies.
**Table S3.** List of Transcription factors that may bind to the 150 bp region flanking rs918980.

## Data Availability

The data that support the findings of this study are available from the corresponding author upon reasonable request.
