# Supplementary material for: Characterisation of the role played by ELMO1, GPR141 and the intergenic polymorphism rs918980 in Fuchs' dystrophy in the Indian population
Source: FEBS Open Bio. 2025 Feb 19;15(5):822–35. doi: 10.1002/2211-5463.70006 (PMC12051025; doi:10.1002/2211-5463.70006)
Supplement: Supplementary file 1 — Fig. S1. Genomic location of SNPs rs918980, rs66496742 and rs6975846. Table S1. List of primers. Table S2. Demographics of the study participants in the genetic association studies. Table S3. List of Transcription factors that may bind to the 150 bp region flanking rs918980. [file FEB4-15-822-s001.docx]

**Supplementary Materials:**

**Supplementary Table 1: List of primers**

Forward and reverse primer sets used in the genotyping of the SNP rs918980, and its LD SNP rs6975846, PCR amplification for luciferase assay with rs918980, and qRT PCR of ELMO1 and GPR141 genes and internal control Beta- Actin.

| Sl number | Primer name | Forward Primer |
| --- | --- | --- |
| 1 | rs918980 PCR forward | 5’ ATGCCTTTGTTCCCCTATTTTCAG 3’ |
| 2 | rs918980 PCR reverse | 5’ AACCCTTGTTACCTCTGTTTCATC 3’ |
| 3 | rs6975846 PCR forward | 5’TTGGGCAGGCAACTTCTAAATCA3’ |
| 4 | rs6975846 PCR reverse | 5’TTTCACGCTGTATGCTGTCTCC3’ |
| 5 | rs918980_luciferase_PCR forward | 5'GCGGGGTACCACCAAATGTAGCTGGGGAAGAA3’ |
| 6 | rs918980_luciferase_PCR reverse | 5'TAAACTCGAGTGGAGTACCAGGACAAACAAAC3’ |
| 7 | ELMO1 qRT_forward | 5’GTGATGGGTGGTCTCTTGCC3’ |
| 8 | ELMO1 qRT_reverse | 5’TTCTGGTATCGCTCAGTGCC3’ |
| 9 | GPR141 qRT_forward | 5’TTACGGTGACTTCCCAAGTAT3’ |
| 10 | GPR141 qRT_reverse | 5’TCCATACCGGGAGACAACCA3’ |
| 11 | Beta-actin qRT forward | 5’CACTCTTCCAGCCTTCCTTC3’ |
| 12 | Beta-actin qRT reverse | 5’GTACAGGTCTTTGCGGATGT3’ |

**Supplementary Table 2: Demographics of the study participants in the genetic association studies**

To rule out the age and gender biases of the study, a t-test was performed between the age of individuals in the control and FECD patient groups as well as between the total number of males and females in each group. The result showed no significant demographic differences between the two groups.

| **Parameters** | **Control** | **FECD** | **P value** |
| --- | --- | --- | --- |
| Sample size | 379 | 128 |  |
| Mean age ± SD (Age) | 60.56 ± 8.7 | 59.65 ± 9.9 | 0.35 |
| Male: Female | 1: 1.78 | 1: 2.15 | 0.49 |

**Supplementary Table 3: List of Transcription factors that may bind to the 150bp region flanking rs918980**

In-silico analysis resulted in a total number of 10 transcription factors, which have an affinity to bind to the 150bp region surrounding the SNP rs918980. MSX1 and LHX 9 showed to have the highest affinity.

| **Matrix ID** | **Name** | **Score** | **Relative score** | **Start** | **End** | **Strand** | **Predicted sequence** |
| --- | --- | --- | --- | --- | --- | --- | --- |
| [**MA0666.1**](https://jaspar.genereg.net/matrix/MA0666.1) | MSX1 | 11.73 | 1.00 | 85 | 92 | - | CCAATTAG |
| [**MA0701.2**](https://jaspar.genereg.net/matrix/MA0701.2) | LHX9 | 13.11 | 1.00 | 67 | 74 | + | CTAATTAA |
| [**MA0075.3**](https://jaspar.genereg.net/matrix/MA0075.3) | PRRX2 | 12.98 | 1.00 | 67 | 74 | + | CTAATTAA |
| [**MA0716.1**](https://jaspar.genereg.net/matrix/MA0716.1) | PRRX1 | 11.49 | 1.00 | 67 | 74 | + | CTAATTAA |
| [**MA0721.1**](https://jaspar.genereg.net/matrix/MA0721.1) | UNCX | 10.94 | 1.00 | 67 | 74 | + | CTAATTAA |
| [**MA0654.1**](https://jaspar.genereg.net/matrix/MA0654.1) | ISX | 12.07 | 1.00 | 67 | 74 | + | CTAATTAA |
| [**MA0675.1**](https://jaspar.genereg.net/matrix/MA0675.1) | NKX6-2 | 10.87 | 0.99 | 67 | 74 | + | CTAATTAA |
| [**MA0618.1**](https://jaspar.genereg.net/matrix/MA0618.1) | LBX1 | 12.97 | 0.99 | 67 | 74 | - | TTAATTAG |
| [**MA0056.1**](https://jaspar.genereg.net/matrix/MA0056.1) | MZF1 | 9.085 | 0.99 | 13 | 18 | + | TGGGGA |
| [**MA0056.1**](https://jaspar.genereg.net/matrix/MA0056.1) | MZF1 | 9.085 | 0.99 | 78 | 83 | - | TGGGGA |

**Supplementary figure 01:**


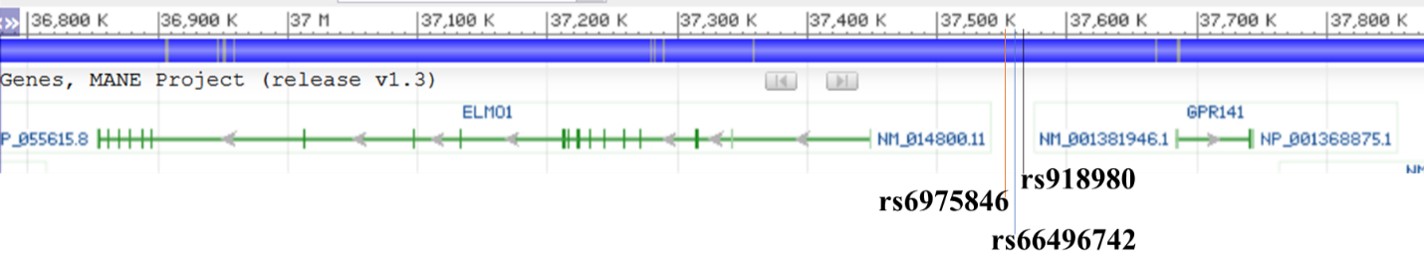


**Supplementary Figure. 1:** NCBI database shows the SNPs rs918980, rs66496742, and rs6975846 are positioned between *ELMO1* and *GPR141* genes.
